# Supported Cu^0^ nanoparticles catalyst for controlled radical polymerization reaction and block-copolymer synthesis

**DOI:** 10.1038/s41598-017-10760-w

**Published:** 2017-09-04

**Authors:** Aurel Diacon, Edina Rusen, Alexandra Mocanu, Leona Cristina Nistor

**Affiliations:** 1 Department of Bioresources and Polymers Science, University Politehnica of Bucharest, 1-7 Gh. Polizu Street, 011061 Bucharest, Romania; 2 Department of Chemical and Biochemical Engineering, University Politehnica of Bucharest, 1-7 Gh. Polizu Street, 011061 Bucharest, Romania; 3 National Institute of Materials Physics, 405A Atomistilor, 077125 Magurele-Ilfov, Romania

## Abstract

The synthesis of Cu^0^ nanoparticles on different supports and their activity in controlled living radical polymerization processes is presented. The type of support influences the final size of the copper nanoparticles as well as their adhesion to the support. These aspects have a direct influence on the characteristics of the polymers obtained. The best results were obtained for SiO_2_ particles, which afforded a good molecular weight distribution (Mw/Mn = 1.25). The activity, recovery and recycling of the catalyst was explored for ultrafast polymerization reaction of butyl acrylate. Further, the terminal bromine reactivity was used for the synthesis of a block poly(n butyl acrylate-block-styrene). The influence of ligand type on the control of the reaction was studied. Also, a straightforward polymerization procedure without any ligand afforded a polydispersity value of 1.38.

## Introduction

Controlled living radical polymerization techniques represent a very appealing research topic and versatile tools for industry, as they allow access to polymers with precise and tailored chemical compositions and architectures^1^. During the last 20 years, the mechanisms and reaction conditions for living radical polymerization have been the subject of extensive studies, resulting well-established techniques such as reversible addition-fragmentation chain transfer polymerization (RAFT)^2, 3^, nitroxide mediated polymerization (NMP)^4^ and atom transfer radical polymerization (ATRP)^5^.

ATRP is a redox catalytic process in which an alkyl halide is usually activated by a transition metal catalyst in a lower oxidation state through the formation of the corresponding alkyl radical and the transition metal in its higher oxidation state. The controlled or “living” characteristic of the process arises from the intermittent and repeated activation/deactivation characteristic of the cycles which leads to the same growth rate for the majority of the polymer chains^5^.

Despite the ATRP process versatility, several key characteristics still need improvement, these include the air sensitivity of the reaction^6^, the recovery and recycling of the catalyst, as well as the transition metal content in the final polymer which leads to its coloration^7, 8^. Solutions for these issues consisted in: the use of different techniques for the catalyst recovery and the development of techniques that limit the amount of copper halides needed for the reaction through the regeneration of catalyst active species using an external agent^9^ or electrochemical methods^10^, the use of supported catalysts^11–21^ and the development of metal free ATRP reaction^22^.

The use of Cu^0^ was explored by several groups with different aims such as: sacrificial agent for catalyst Cu^I^ reactivation^23, 24^, catalyst for development ultrafast reactions^25–27^ for ultrahigh molecular weight polymers at room temperature^26^, the synthesis of well-defined structures^28^, or in the aqueous controlled polymerization for high end-chain fidelity and controlled structure^29^.

The forms in which Cu^0^ was used varies considerably, from the most popular Cu^0^ wire^30^, to copper powder^31^, *in situ* generated Cu^0^ nanoparticles^27, 32^ and *ex situ* generated Cu^0^ nanoparticles^33^ and 1 penny copper coin^34^. The size of the Cu^0^ particles was found to greatly influence the kinetics of the reaction a decrease in their size affording an increase of one order of magnitude of the apparent polymerization rate constant $({k}_{p}^{{app}})$ ^35^.

This study presents the synthesis of catalysts based on copper nanoparticles immobilized on different supports such as silica, titania and alumina employed in the initiation of controlled polymerization reactions. The novelty of our approach consists in the use of *ex situ* synthesized supported copper nanoparticles with high surface area and demonstrated reusability. The size of copper nanoparticles and support type controls the efficiency of the immobilization on the support. These parameters affect also the polymerization reaction by modifying both the molecular weight attained and polydispersity index. The activity, recovery and reuse of the catalyst was explored for butyl acrylate ultrafast polymerization reaction. Standard polymerization reaction conditions involving Me_6_TREN as ligand agent (without Cu^II^ salts), were used to study the catalysts. The reaction in the absence of any ligand was also studied. The catalyst, and synthesized polymer were employed the synthesis of a block-copolymer. The kinetics of the ligand free polymerization reaction was studied in order to calculate the ${k}_{p}^{{app}}$ and to establish whether the process has a “living” characteristic.

## Materials

The monomers butyl acrylate (BA) (Sigma-Aldrich), styrene (ST) (Sigma-Aldrich) were purified by vacuum distillation. Sodium borohydride (NaBH_4_), isopropyl alcohol (IPA), tris[2-(dimethylamino)ethyl]amine (Me_6_TREN), ethyl α-bromoisobutyrate (R-Br), ascorbic acid, dimethylsufoxide (DMSO), CuSO_4_•5H_2_O, tetraethyl orthosilicate (TEOS), sodium dodecylsulfate, cetyltrimethylammonium bromide (CTAB), NaOH from Sigma Aldrich were used without any prior purification.

## Methods

### Nanoparticles synthesis

**The SiO**_**2**_ **particles** were synthesized according to a modified Ströber method^36^. Briefly, 0.22 g CTAB were dissolved in 90 mL H_2_O, followed by the addition of 0.7 mL NaOH 2 M. The solution was stirred at 80 °C for 30 minutes prior to the addition of 0.9 mL of TEOS, and then stirring at 80 °C was continued for 2 h.

**The CuNPs/SiO**_**2**_ **catalyst** was prepared by the dissolution of (0.15 g or 0.3 g) CuSO_4_•5H_2_O in the SiO_2_ dispersion (90 mL) which was further subjected to sonication (using an ultrasonic processor Heischler UP50H) prior (for 20 minutes) and during the reduction process (for 30 minutes) which was performed by using an equimolecular amount of ascorbic acid. The mixture was then stirred at 80 °C for 2 h. The catalyst was separated through centrifugation, washed with water several times and dried under vacuum. The amount of CuSO_4_•5H_2_O was varied in order to obtain two CuNPs/SiO_2_ catalysts containing 18%, respectively 36% (weight %) Cu^0^.

**The CuNPs/Al**_**2**_ **O**_**3**_ and the **CuNPs/TiO**_**2**_ were prepared by dispersing 0.25 g of support (aluminum oxide 90 active neutral_;_ or titanium dioxide P25) in 90 mL H_2_O with 0.1 g CTAB and 0.7 mL NaOH 2 mol/L. To the support dispersion were then added 0.15 g CuSO_4_•5H_2_O. This solution was sonicated for 20 minutes prior to the addition of ascorbic acid (stoichiometric vs copper salt) and continued for another 30 minutes. The mixture was stirred for 2 h at 80 °C. Then, the catalyst was separated through centrifugation, washed with water several times and dried under vacuum.

The polymerization procedure was based on literature reports for ultrafast SET-LRP reaction^25, 30^. Briefly, in a reaction vial were introduced 4 mL of BA and 1.4 mL of IPA. The mixture was then purged with nitrogen for 5 minutes, after which 30 µl ethyl α-bromoisobutyrate, 20 mg amine (Me_6_TREN or BiPy), 25 mg Cu catalyst, 3 mg NaBH_4_ and 0.6 mL H_2_O were introduced in the reaction vial and the reaction was kept under stirring for 30 minutes. The reaction mixture was precipitated in methanol, centrifuged and dried until a constant mass was obtained. In short, the reaction conditions were [BA]_0_: [R-Br]_0_: [Cu^0^]_0_: [Me_6_TREN]_0_: [NaBH_4_]_0_ = 137: 1: 0.35: 0.41: 0.35.

For the **recovery of the catalysts**, the polymerization reaction mixture was centrifuged, the catalyst being then dispersed in DMSO and centrifuged several times, and dried under vacuum.

The **block copolymer** synthesis was realized by the addition of 1 mL styrene and 3 mL DMSO to the BA polymerization reaction mixture. Nitrogen was introduced in this new mixture and the reaction was activated by addition of 3 mg of NaBH_4_. The reaction mixture was stirred for 2 h, after which it was precipitated in methanol, filtrated and dried until a constant mass was attained.

### Characterization

The molecular weights of the resulted polymers and oligomers were analyzed using PL-GPC 50 Integrated GPC/SEC System (Agilent Technologies) using a 1 mL/min THF flow rate and a column oven temperature of 30 °C using polystyrene as standard. The GPC traces were treated according to Gavrilov *et al*.^37^.

The high-resolution transmission electron microscopy (HRTEM) studies were performed on an atomic resolution analytical JEOL JEM-ARM 200 F electron microscope, operating at 200 kV. Specimens for HRTEM were prepared in the following way: a drop of the solution was put on holey carbon TEM grid and dried at 100 °C for 5 min.

## Results and Discussion

Currently there is still a real controversy regarding the atom transfer radical polymerization (ATRP) between the single electron transfer living radical polymerization SET-LRP^25, 38^ and supplemental activator and reducing agent SARA-ATRP^39–42^.

This study presents an ATRP polymerization process starting from CuNPs deposited on different supports with or without the presence of complexing amine species. The aim is to present a catalyst that can be recycled and to bring more information related to the mechanism of the reaction.

Considering that the SET-LRP^26^ mechanism implies Cu^0^ species as the activating agent, the first approach of this study consisted in the synthesis and characterization of a catalyst comprised of copper nanoparticles generated on the surface of SiO_2_ particles. The aim of the synthesis is to obtain a catalyst with high surface area able to initiate a SET-LRP process and also allows its recovery, re-activation and re-utilization.

**Figure 1:**
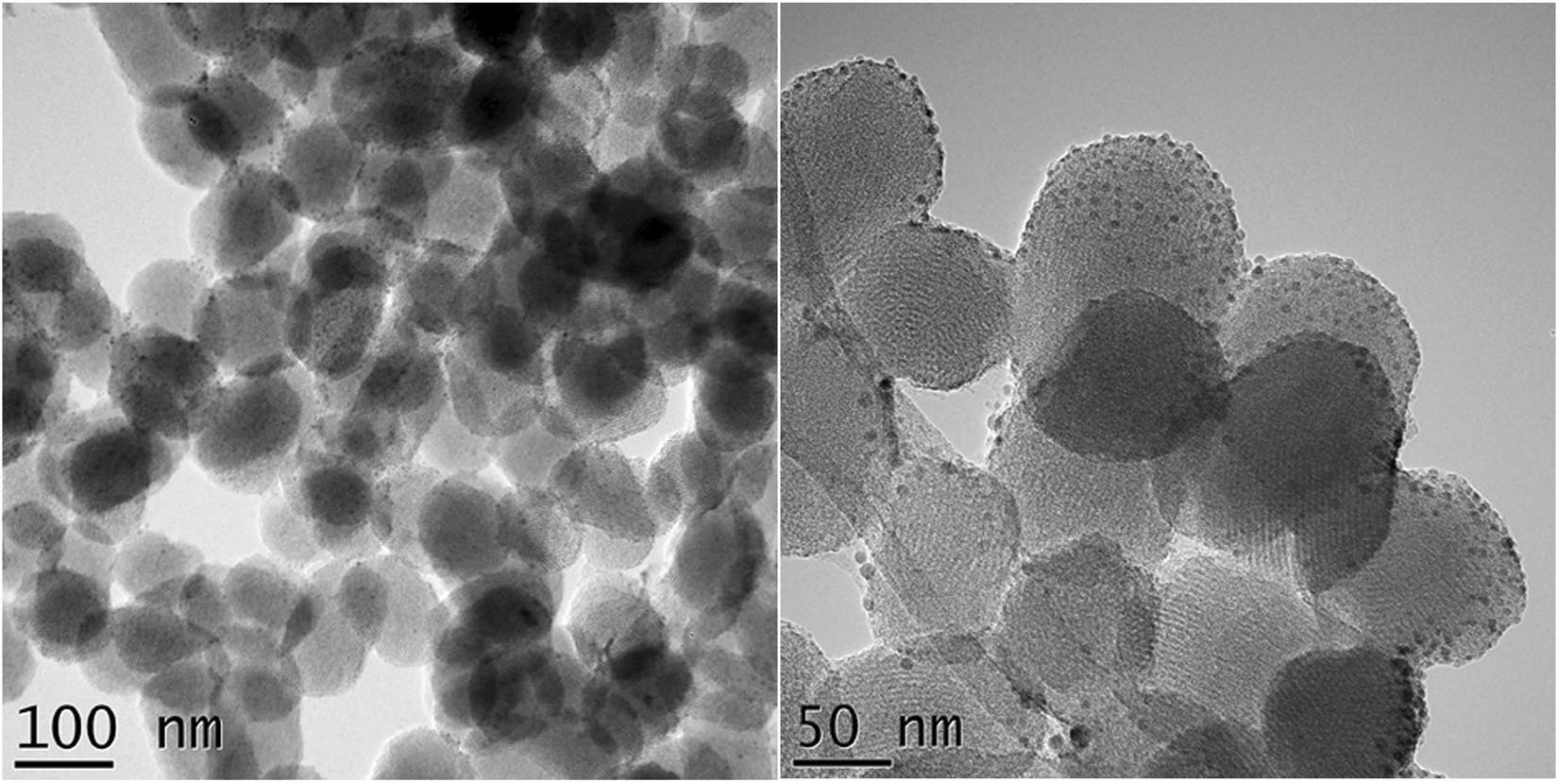
TEM images of the CuNPs/SiO_2_ catalyst at different magnifications.

In order to characterize the morphology of the CuNPs/SiO_2_ catalyst, transmission electron microscopy (TEM) analyses were employed. In Fig. 1, one can observe that on the surface of the SiO_2_ particles with ellipsoidal shapes, and sizes around 100 nm, the Cu nanoparticles with dimensions smaller than 10 nm are uniformly spread. Some of the silica particles present the characteristic mesoporous structure.

**Figure 2:**
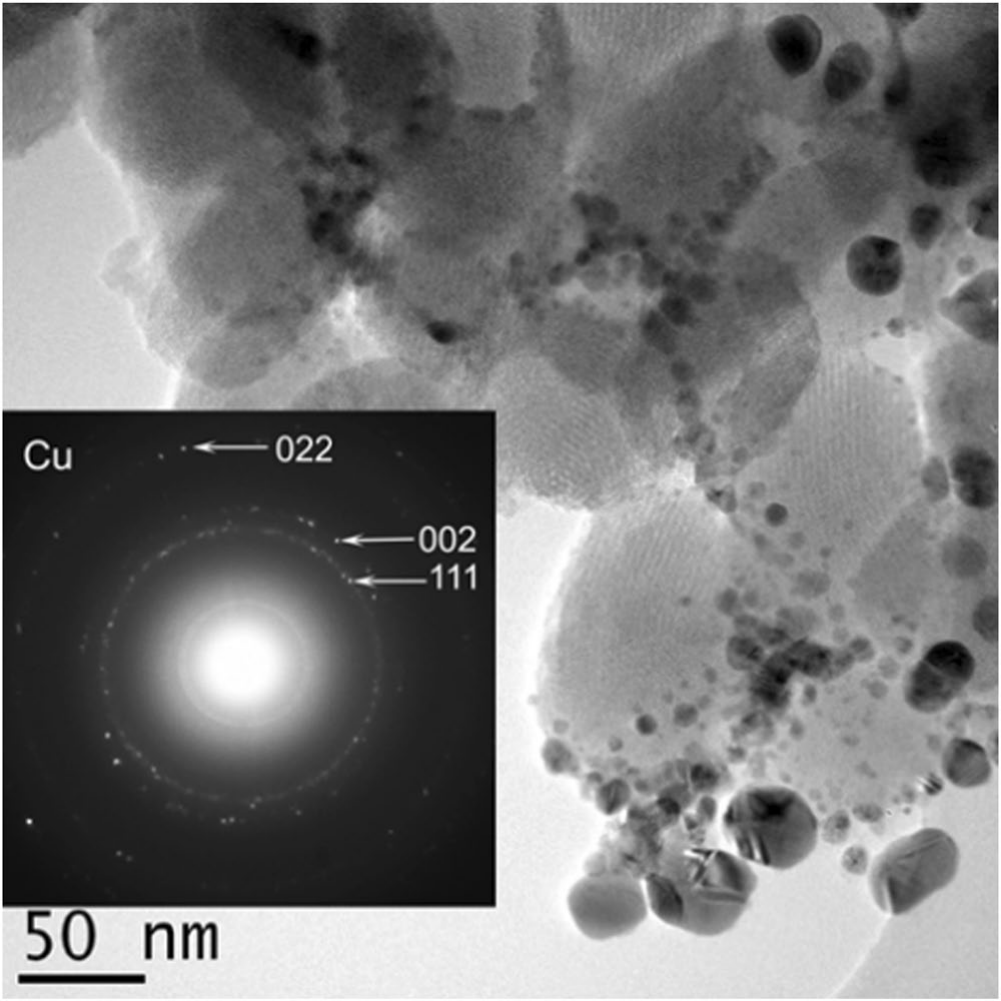
TEM images for CuNPs/SiO_2_ catalyst and electron diffraction pattern (insert).

In order to confirm the oxidation state of the CuNPs, Fig. 2 shows a region of the specimen where larger Cu nanoparticles where also present. Here it was possible to obtain an electron diffraction pattern (insert of Fig. 2) which was indexed with the metallic copper (Cu^0^) structure. Therefore, the nanoparticles are in a reduced state. This aspect suggests that we can use the catalyst for the initiation of the polymerization reaction in a SET-LRP mechanism.

As presented in the literature^25, 30, 38, 43^, the polymerization initiation requires activation with NaBH_4_ for the generation of the SET-LRP active species. The high specific surface area of our catalyst compared to literature examples such as Cu wire, allows a fast reaction. Therefore, a conversion of 90% is reached in 30 minutes. In the SET-LRP mechanism, the dominant reactions are the activation of alkyl halides by Cu^0^, the deactivation of radicals by Cu^II^ and the disproportionation of Cu^I^ species to regenerate Cu^0^ and Cu^II^. In the SET-LRP reaction, there is minimal activation of alkyl halides by Cu^I^, due to an instantaneous disproportionation, and negligible deactivation of radicals by Cu^I^ or comproportionation.

**Figure 3:**
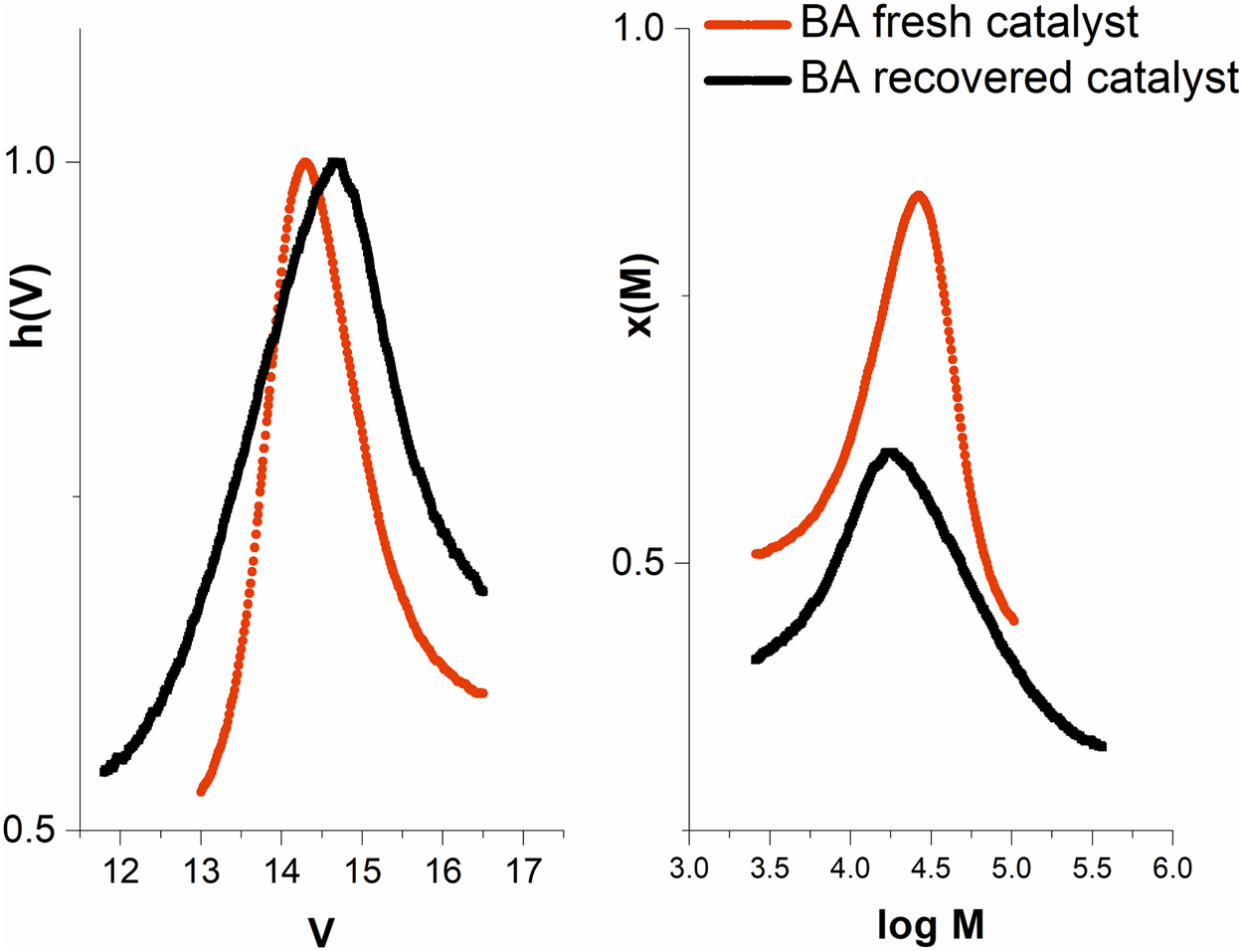
GPC traces for BA-fresh catalyst and BA-recycled catalyst.

The use of a catalyst with high surface area, easily separable and reusable can facilitate the scale-up of the process. To this end, we have recovered the catalyst after the first reaction and recycled it in another polymerization step under the same conditions. The GPC analyses of the first and second BA polymerization experiment are presented in Table 1 and Fig. 3.

**Table 1:** GPC analysis – BA polymerization with catalyst recycling.

	Peak	Mn (g/mol)	PD	Time	Conv. (%)	Mn th. (g/mol)
CuNPs/SiO_2_ first use	1	21300	1.25	30 min	90	50100
CuNPs/SiO_2_ second use	1	21300	1.39	30 min	88	50100

The comparison of the molecular weights obtained reveals only a slight change of the values for the second experiment which afforded a lower molecular weight and a larger polydispersity (PD). This change can be explained by the increase of the surface area due to the consumption of Cu^0^ which leads to higher number of active sites. Thus, the lower molecular weight and the increased polydispersity of the second experiment, can both be explained by an increased number of active sites in the case of the recovered catalyst.

**Figure 4:**
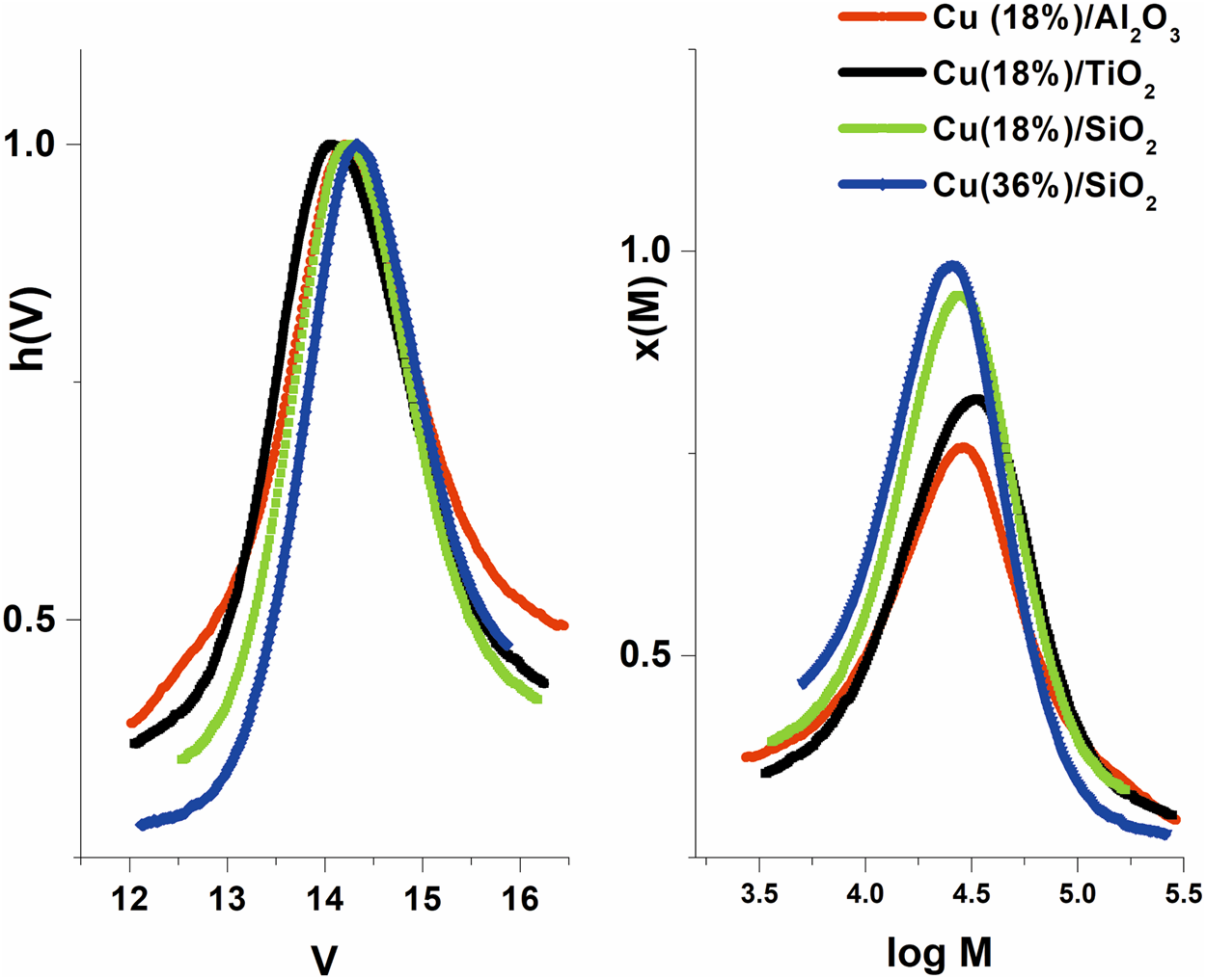
GPC traces for poly(butyl acrylate) obtained using different catalysts using Me_6_TREN ligand.

The support can play an active role in heterogeneous catalysis; therefore, we have synthesized copper nanoparticles on different supports and at different level of concentration. We have opted for Al_2_O_3_ and TiO_2_ as supports in addition to the SiO_2_ particles. All the supports have in common the presence of hydroxyl functional groups at the particle surface, which can interact with the copper ions prior to the reduction process and facilitate the nucleation and adhesion of the Cu nanoparticles. Also, we have prepared a catalyst on SiO_2_ particles with a double the amount of CuNPs (weight %) in order to study the influence copper concentration. The GPC analyses of the polymers obtained using the different catalysts are presented in Table 2 and Fig. 4.

**Table 2:** GPC analyses results - Influence of the catalyst type on the polymerization reaction, using Me_6_TREN ligand.

Catalyst	Mn (g/mol)	PD	Time	Conv. (%)	Mn th. (g/mol)
CuNPs/Al_2_O_3_ (18% Cu, weight %)	19100	2.12	30 min	67	50100
CuNPs/TiO_2_ (18% Cu, weight %)	23600	1.54	30 min	80	50100
CuNPs/SiO_2_ (18% Cu, weight %)	21300	1.25	30 min	90	50100
CuNPs/SiO_2_ (36% Cu, weight %)	18700	1.48	30 min	95	25500

The analysis of Table 2 and Fig. 4 reveals that the molecular weight is only slightly influenced by the support. In order to investigate these characteristics TEM analysis was used to characterize each catalyst (see Supplementary Information Figs S1–S3). The TEM analysis of CuNPs/Al_2_O_3_ catalyst (See Supplementary Information Fig. S1) revealed a high dimensional polydispersity of the CuNPs with a considerable amount of CuNPs (larger than 30 nm), which do not adhere to the support. These aspects, can justify the PD value of the GPC analysis, if we consider the different reactivity of the active sites on small and larger particles.

In the case of CuNPs/TiO_2_ catalyst, the increase of the PD value could be attributed to a lower adhesion of CuNPs to the TiO_2_ nanoparticles, which is suggested by the presence of CuNPs separated from the support (See Supplementary Information Fig. S2). Further, the smaller CuNPs particle size compared with the CuNPs/Al_2_O_3_ catalyst should result in a lower molecular weight due to the increased surface area. However, rather similar molecular weights were registered.

In SET-LRP mechanism, the Cu^0^ concentration plays a crucial role. In order to explore this aspect, we have synthesized a CuNPs/SiO_2_ with an increased quantity of copper. From Fig. 2 it can be observed that the molecular weight slightly decreases, probably due to the increase in the active centers, in accordance with the increased concentration of Cu^0^ on the surface of the support. For the CuNPs/SiO_2_ (36% weight - Cu) catalyst, the TEM analysis revealed the presence, besides the small nanoparticles, of very large (~100 nm) particles (See Supplementary Information Fig. S3). The overall size of the CuNPs is smaller compared to the first catalyst CuNPs/SiO_2_ (18% Cu, weight%), but the presence of the very large particles should explain the similar GPC results. Nevertheless, the difference between the two CuNPs/SiO_2_ catalysts lies in the conversion attained at the same interval. The increase in copper content afforded a slightly higher conversion. However, the modification is not proportional to the increase of the surface area. A possible explanation could be the limitation imposed the diffusion/dissolution process of copper species (Cu^I^ and Cu^II^) into the reaction medium.

Another aspect that needed to be ascertained was the reactivity of the terminal bromine of the obtained poly(butyl acrylate). Thus, styrene was used to present the successful synthesis of a BA-ST block-copolymer. This choice is motivated by the possible commercial applications of the BA-ST block-copolymers^44, 45^.

**Figure 5:**
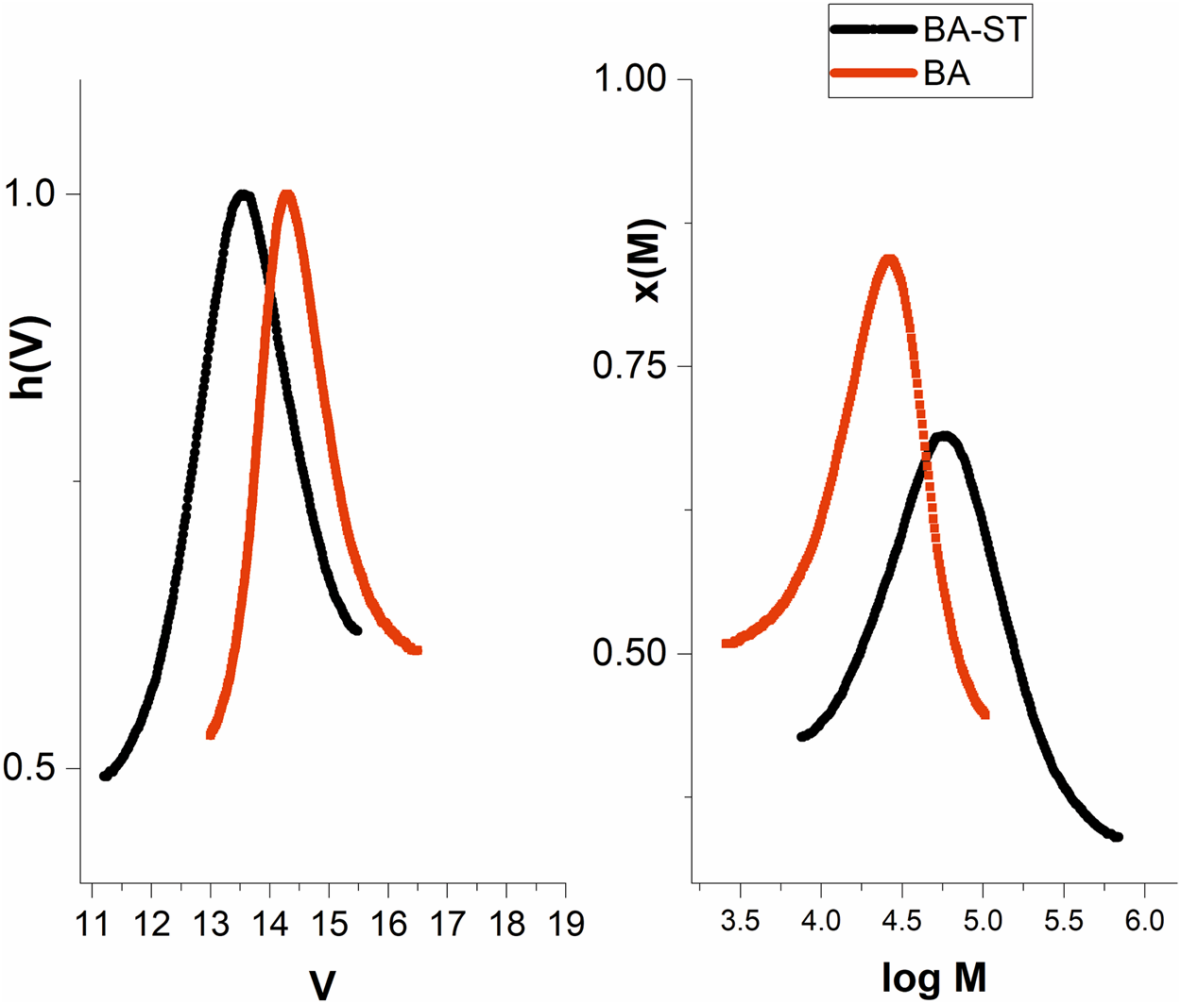
GPC traces for block poly(n butyl acrylate-block-styrene) synthesized using CuNPs/SiO_2_ catalyst.

Table 3 and Fig. 5 demonstrate the block-copolymer synthesis evidenced by the increased molecular weight obtained.

**Table 3:** GPC data for block poly(n butyl acrylate-block-styrene) obtained using CuNPs/SiO_2_ catalyst and Me_6_TREN ligand.

Cat. CuNPs/SiO_2_	Peak	Mn (g/mol)	PD	Time	Conv. (%)	Mn th. (g/mol)
PolyBA	1	21300	1.25	30 min	90	50100
PolyBA-ST	1	43700	1.72	120 min	30	25600

**Figure 6:**
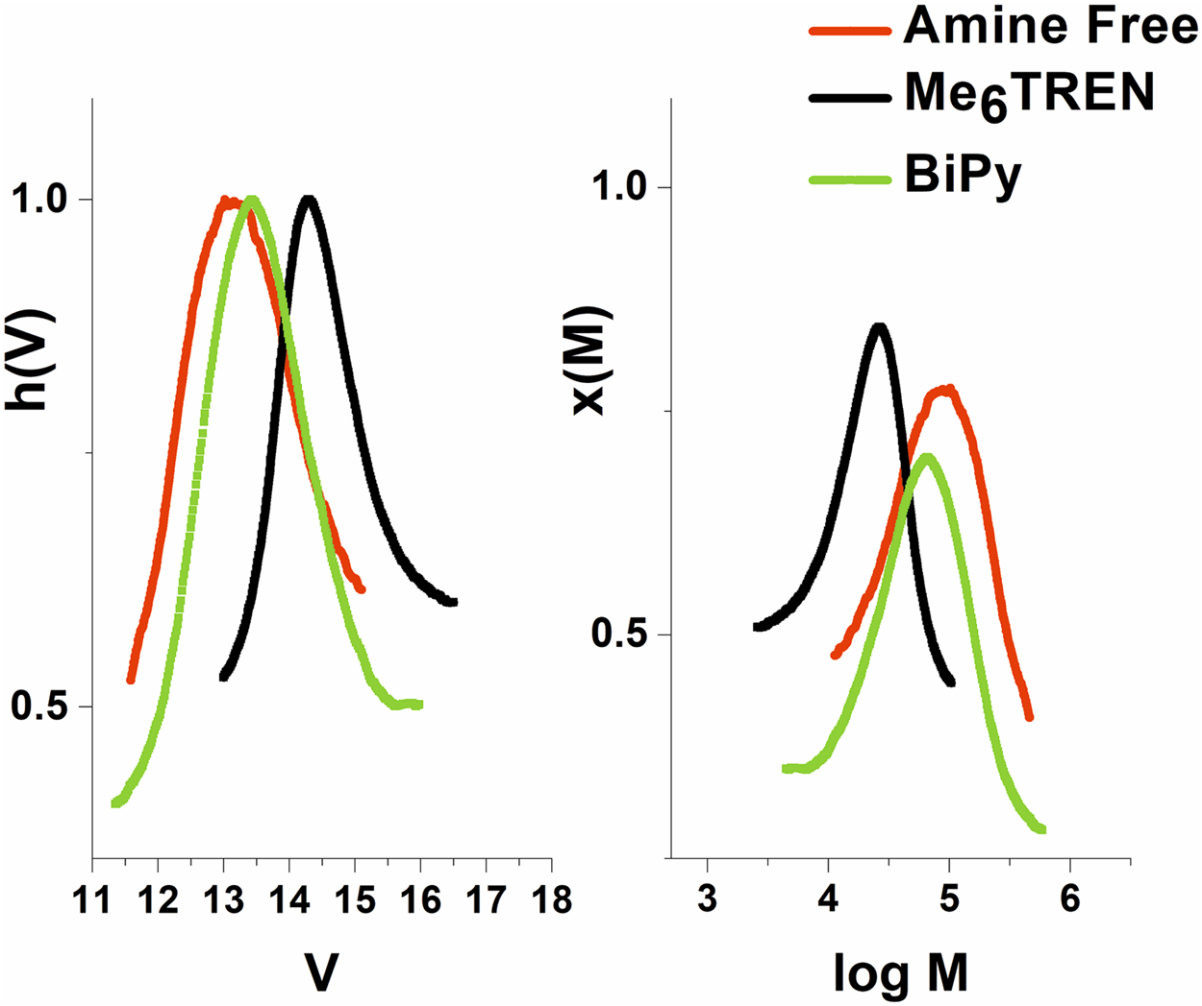
GPC analyses for BA polymerization without any ligand, with Me_6_TREN and with BiPy.

The SET-LRP mechanism involves the CuNPs, but it is also dependent on the concentrations of Cu^I^ and Cu^II^ through disproportionation reaction. As the experiments revealed, an increased copper concentration did not lead to significant changes in the reaction result, which constitutes an indirect evidence that the reaction follows a SET-LRP mechanism. It is interesting to follow the evolution of the reaction in the absence of complexing ligands or using different agents, because the disproportionation reactions will be affected and the reaction could involve only CuNPs as Cu^0^ source if no ligand is used. Therefore, the polymerization was realized in the absence of Me_6_TREN and in the presence of BiPy. As can be observed in Table 4 and Fig. 6, the lowest molecular weight was obtained in the case of Me_6_TREN, which can be explained by the participation of Me_6_TREN both in disproportionation and in polymer chain transfer reactions^46^. In contrast, the highest molecular weight was registered in the absence of any ligand, whereas in the case of BiPy an intermediary molecular weight was registered due to its participation in disproportionation reaction with limited chain transfer reactions.

**Table 4:** GPC data for BA polymerization using CuNPs/SiO_2_ and different ligand agents.

Ligand Agent	Mn (g/mol)	PD	Conv, at 30 min	Mn th. (g/mol)
Me6TREN, CuNPs/SiO_2_	21300	1.25	85%	50100
No Amine, CuNPs/SiO_2_	70600	1.38	85%	50100
BiPy, CuNPs/SiO_2_	44800	1.73	85%	50100

In the case of BA polymerization in the absence of any ligand agent and using BiPy, the GPC analyses revealed an increased PD value. The results are consistent with literature data which presents the importance of N-ligands^47, 48^ that stabilize Cu^II^ and facilitate the disproportionation of Cu^I^. In our case this behavior explains the narrower PD value for Me_6_TREN due to the increase of Cu^II^ concentration. However, as interesting result, a PD value < 1.4 was obtained for the amine free reaction. Although the control of the reaction is limited, the straightforwardness of the approach would facilitate a scale-up of the process.

**Figure 7:**
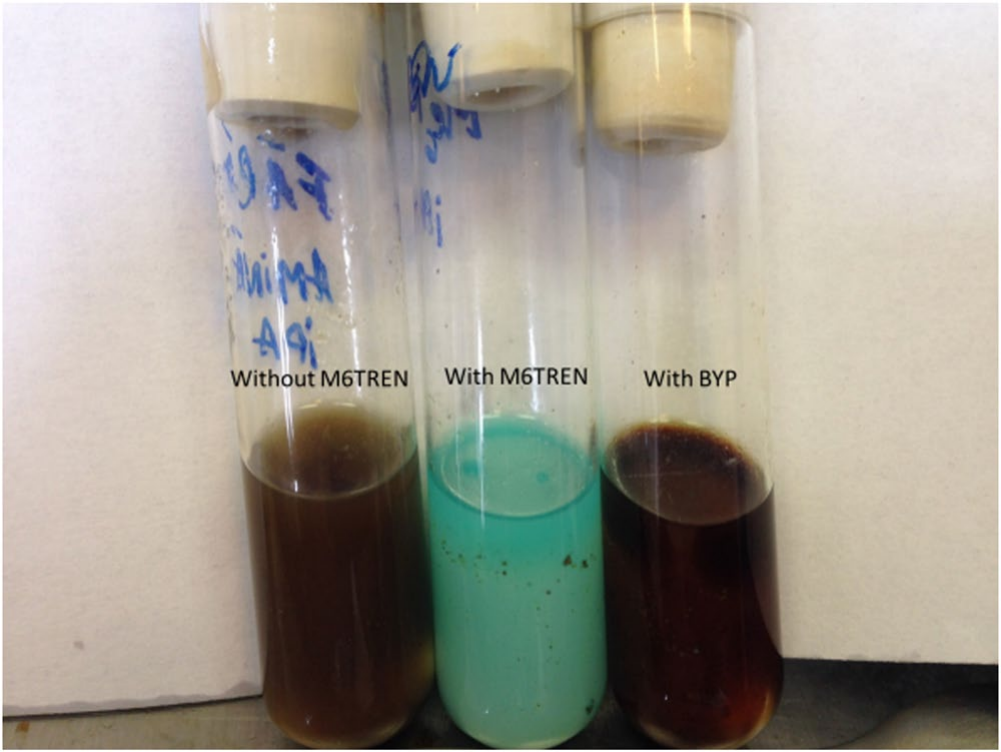
Visualization of BA polymerization using CuNPs/SiO_2_ catalyst and a) no ligand, b) Me_6_TREN and c) BiPy.

In Fig. 7, are presented the characteristic colors resulting during the polymerization process in the absence of ligand agent, using Me6TREN and using BiPy. In the first case, a brown-violet is observed, second blue and in the third case red. These colors confirm the ATRP mechanism for the polymerization, they are specific for the cooper amine complex formed (blue and red), respectively the violet color confirms the Cu^0^ nanoparticles free of ligand.

**Figure 8:**
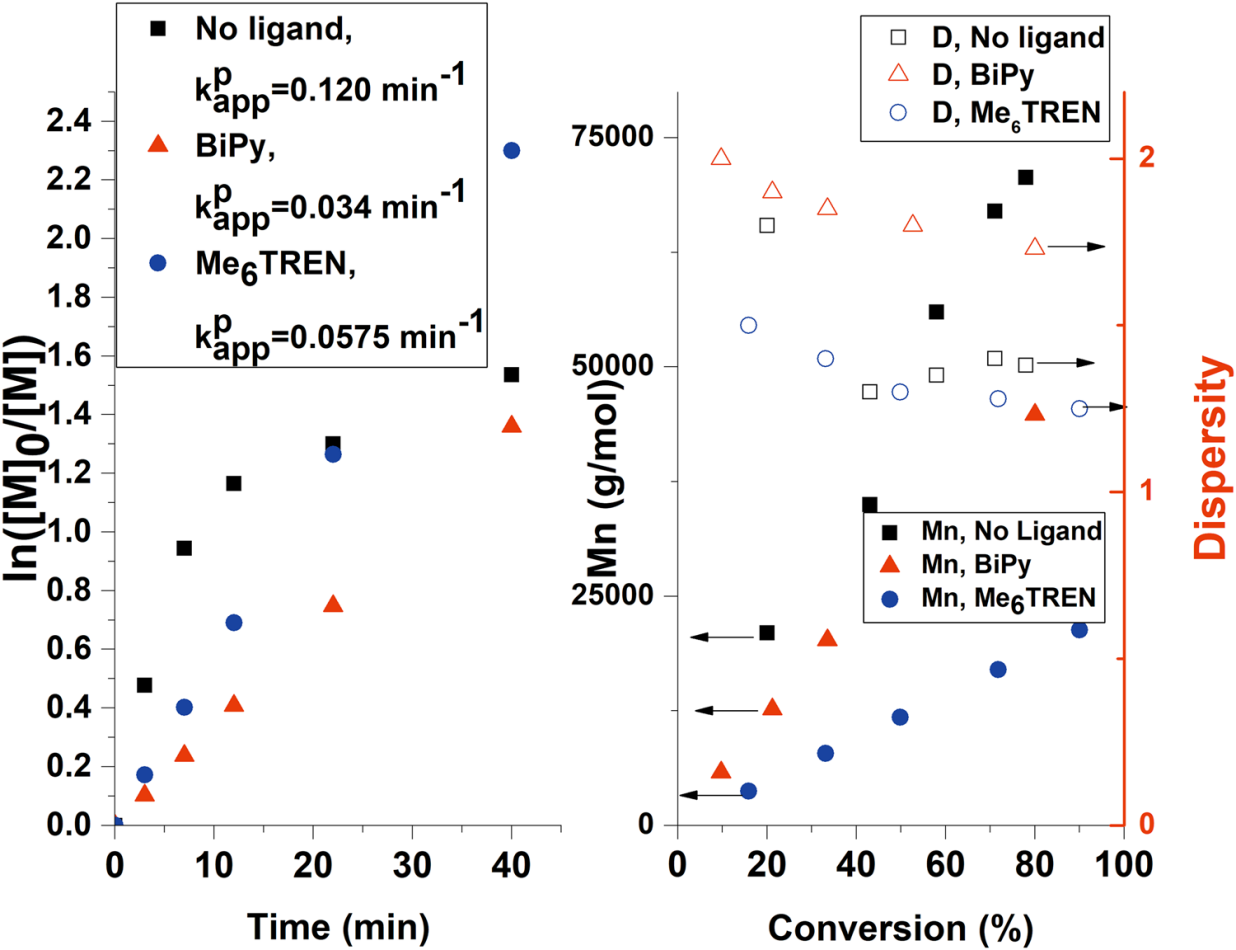
Kinetics plot for BA polymerization using CuNPs/SiO_2_ catalyst and different ligands.

The kinetics analysis of the polymerization reaction in the absence of any ligand was performed and it was compared to the two other cases. Thus, using the plot of ln([M]_0_/[M]) as a function of time (Fig. 8), the ${k}_{p}^{{app}}$ value was calculated as 0.120 min^−1^ (no ligand), 0.0575 min^−1^ (Me_6_TREN) and 0.034 min^−1^ (BiPy), results which are consistent with other examples in the literature for polymerization reactions involving Cu^0^ and Me_6_TREN ligand^34, 35^. In the case of Me_6_TREN there is a linear dependence of M_n_ on the conversion demonstrating the “living” characteristic of the polymerization process. This behavior is manifested only up to 43% conversion in the absence of any ligand. After this point, the reaction involves termination through a chain transfer process or biradical termination.

However, the reasonable 1.38 dispersity value could be explained by the aqueous media employed for the reaction, which allows limited disproportionation and formation of an equilibrium between the copper species.

The obtained results sustain the SET-LRP mechanism for BA polymerization using CuNPs/SiO_2_ catalyst in the presence of Me_6_TREN for the following reasons: (i) the high reducing capacity of NaBH_4_ makes possible the presence Cu^0^ species as active species at the beginning of the reaction, thus, the high reaction rate makes Cu^0^ the activating agent;

(ii) the polymerization rate is dependent on the concentration of Cu^0^ – CuNPs (36%)/SiO2 afforded a 95% conversion at 30 minutes;

(iii) the lack of complexing ligand affords a higher molecular weight confirming limited disproportioning reaction and Cu^0^ as active species;

## Conclusions

Novel catalysts involving CuNPs (Cu^0^) on different supports were synthesized and used in SET-LRP polymerization of BA. The supports consisted in TiO_2_ nanoparticles, SiO_2_ nanoparticles and Al_2_O_3_ particles. The supports afforded under the same reaction conditions different sizes of CuNPs or different adhesion characteristics. The influence of these aspects was correlated with the GPC results for the BA polymerization experiments. The best results in terms of polydispersity were obtained for SiO_2_ particles with a copper concentration of around 18% weight. Further, two consecutive experiments were performed demonstrating its reusability. The reactivity of the terminal bromine was evidenced through the synthesis of BA-ST block copolymer with an increased molecular weight compared to the BA homopolymer.

The control aspect of the reaction relies on the activity of the Me_6_TREN ligand as demonstrated by the experiments using BiPy and no ligand. Interestingly, the reaction performed in the absence of any ligand agent afforded a polydispersity value < 1.4 which sustains a somewhat limited control over the reaction.

Thus, we have shown the synthesis of a reusable catalyst able to participate in controlled living radical polymerization processes (in the presence of Me_6_TREN ligand PD value 1.25) as well as in straightforward polymerization processes with acceptable PD values (PD < 1.4) in the absence of any ligand. These aspects could facilitate the scale-up of the polymerization process.

## Electronic supplementary material

Supplementary information
