# Supplementary material for: Supported Cu0 nanoparticles catalyst for controlled radical polymerization reaction and block-copolymer synthesis
Source: Sci Rep. 2017 Sep 4;7:10345. doi: 10.1038/s41598-017-10760-w (PMC5583343; doi:10.1038/s41598-017-10760-w)
Supplement: Supplementary file 1 — Supplementary information [file 41598_2017_10760_MOESM1_ESM.doc]

**Supported Cu^0^ nanoparticles catalyst for controlled radical polymerization reaction and block-copolymer synthesis**

Aurel Diacon[[1]](#footnote-2), Edina Rusen1,[[2]](#footnote-3)*, Alexandra Mocanu[[3]](#footnote-4), Leona Cristina Nistor[[4]](#footnote-5)


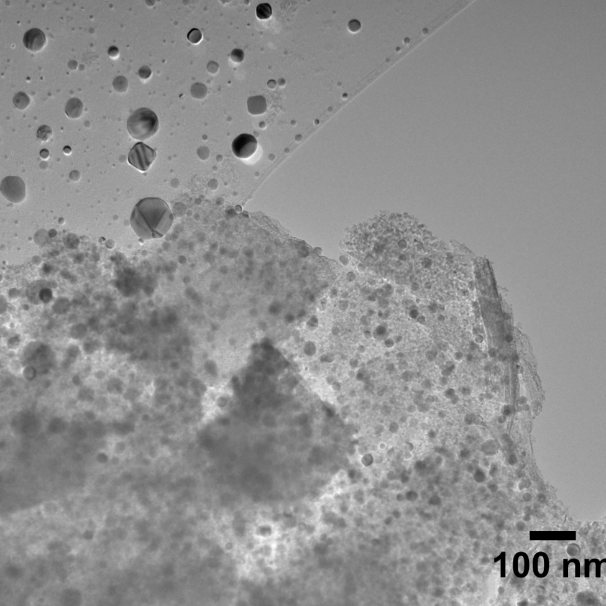

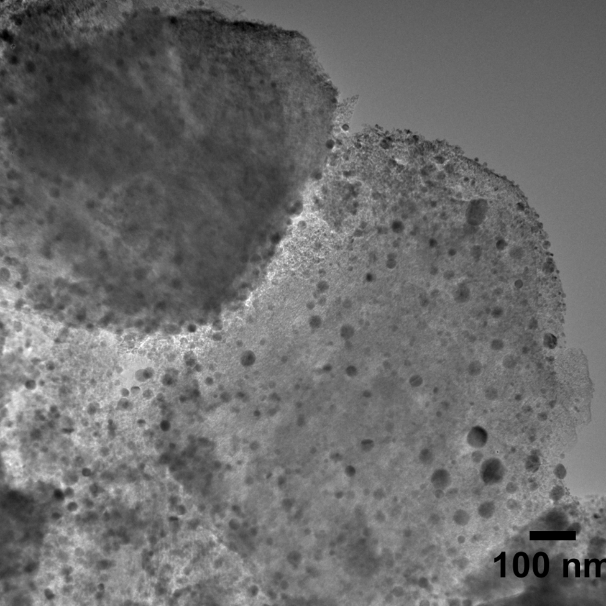


**Fig. S1. TEM images of CuNPs/Al_2_O_3_ catalyst at different magnifications**


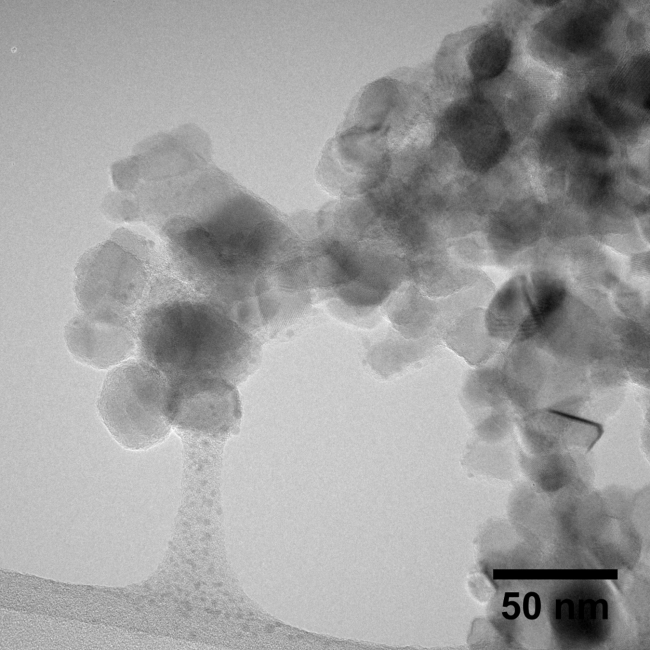

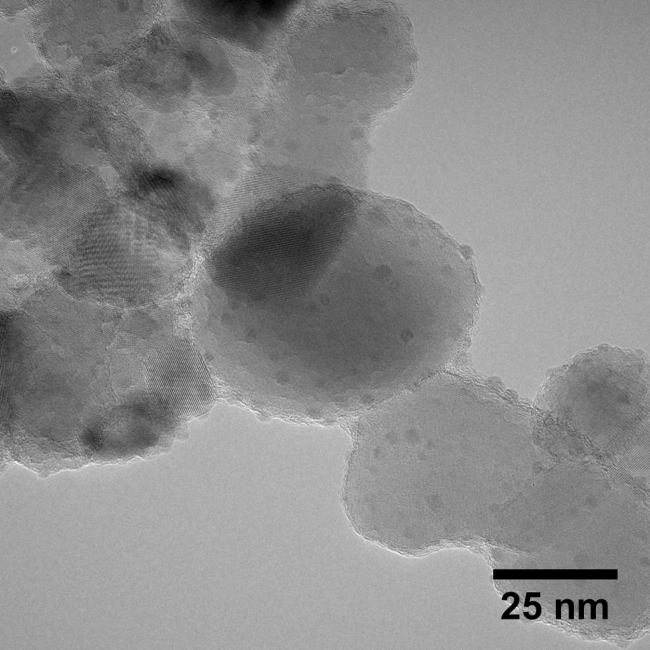


**Fig. S2. TEM images of CuNPs/TiO_2_ catalyst at different magnifications**


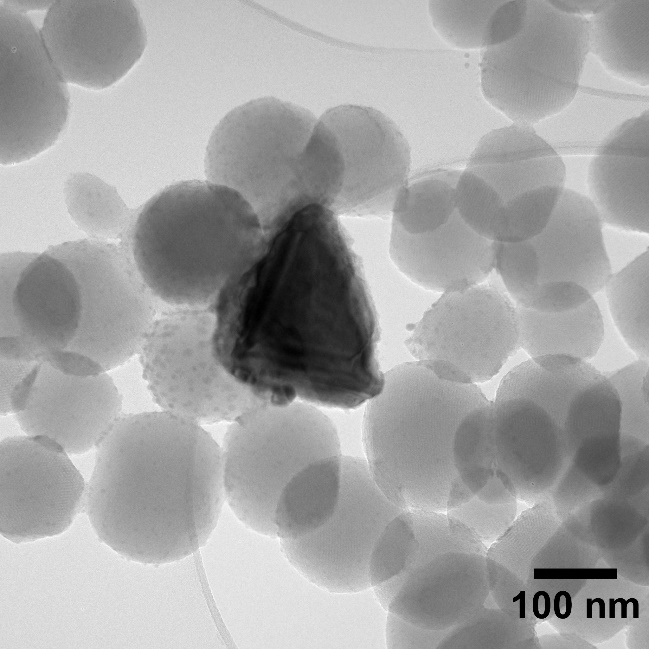

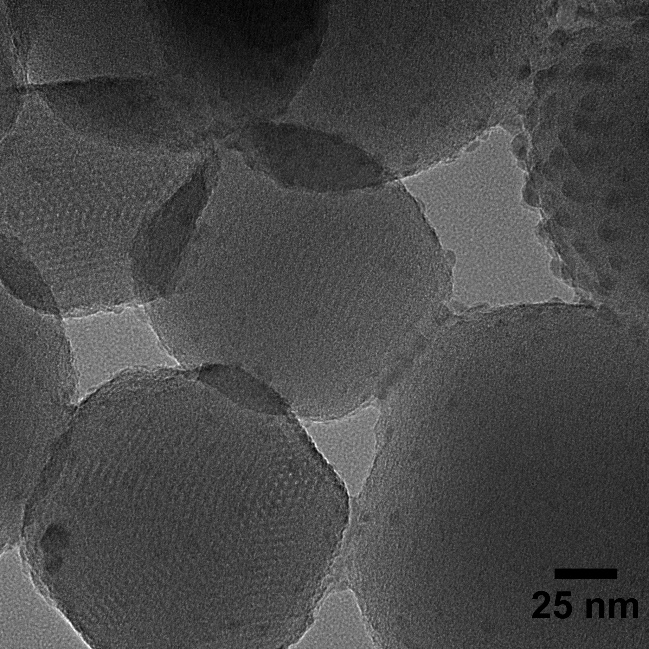


**Fig S3. TEM images of CuNPs/SiO_2_ catalyst (36% Cu weight%) at different magnifications**

1. *Department of Bioresources and Polymers Science, University Politehnica of Bucharest, 1-7 Gh. Polizu Street, 011061 Bucharest, Romania* [↑](#footnote-ref-2)
2. ^^ *Correspondence and requests for materials should be addressed to E.R. (email: edina_rusen@yahoo.com)* [↑](#footnote-ref-3)
3. *Department of Chemical and Biochemical Engineering, University Politehnica of Bucharest, 1-7 Gh. Polizu Street, 011061 Bucharest, Romania* [↑](#footnote-ref-4)
4. *National Institute of Materials Physics, 405A Atomistilor, 077125 Magurele-Ilfov, Romania* [↑](#footnote-ref-5)
